# Rare earth element geochemistry characteristics of seawater and porewater from deep sea in western Pacific

**DOI:** 10.1038/s41598-017-16379-1

**Published:** 2017-11-28

**Authors:** Yinan Deng, Jiangbo Ren, Qingjun Guo, Jun Cao, Haifeng Wang, Chenhui Liu

**Affiliations:** 1 Key Laboratory of Marine Mineral Resources, Ministry of Land and Resources, Guangzhou, 510075 China; 2 Guangzhou Marine Geological Survey, China Geological Survey, Guangzhou, 510075 China; 3 Institute of Geographic Sciences and Natural Resources Research, Chinese Academy of Sciences, Beijing, 100101 China; 4 College of Resources and Environment, University of Chinese Academy of Sciences, Beijing, 100049 China; 5 State Key Laboratory for Mineral Deposits Research, School of Earth Sciences and Engineering, Nanjing University, Nanjing, 210008 China

## Abstract

Deep-sea sediments contain high concentrations of rare earth element (REE) which have been regarded as a huge potential resource. Understanding the marine REE cycle is important to reveal the mechanism of REE enrichment. In order to determine the geochemistry characteristics and migration processes of REE, seawater, porewater and sediment samples were systematically collected from the western Pacific for REE analysis. The results show a relatively flat REE pattern and the HREE (Heavy REE) enrichment in surface and deep seawater respectively. The HREE enrichment distribution patterns, low concentrations of Mn and Fe and negative Ce anomaly occur in the porewater, and high Mn/Al ratios and low U concentrations were observed in sediment, indicating oxic condition. LREE (Light REE) and MREE (Middle REE) enrichment in upper layer and depletion of MREE in deeper layer were shown in porewater profile. This study suggests that porewater flux in the western Pacific basin is a minor source of REEs to seawater, and abundant REEs are enriched in sediments, which is mainly caused by the extensive oxic condition, low sedimentation rate and strong adsorption capacity of sediments. Hence, the removal of REEs of porewater may result in widespread REE-rich sediments in the western Pacific basin.

## Introduction

The rare earth element (REE) is important not only in world industrial demand^1^, but also in tracing various geochemical processes^2^. REE is widely used for geochemical studies of estuaries, oceans and hydrothermal processes^3–17^. The main sources of REEs to seawater are the riverine flux^18–20^ and the eolian flux^21^. The fact that removal of REEs tend to occur rapidly at the low salinity end of the mixing zone, reflecting REE scavenging by salt-induced coagulation and flocculation of Fe-rich, organic colloids^22^. The order of removal is LREE > MREE > HREE^2^. In this case, LREE and MREE with high mobility are preferentially absorbed onto particulates and removal from seawater^23^. REE mainly exist as carbonate complexation with positive or negative charge^24,25^, and the intensity of complexes improve following the increase of the atomic number^25,26^. At higher salinities the particles with strong REEs enrichment release the REE back to solution^10,22^.

The seawater REE cycle, however, has not been fully understood yet^27,28^. Johannesson *et al*.^29^ proposed that groundwater from seafloor would input abundant REEs into seawater, representing a “new” REEs inputs to seawater. There are also a lot of investigations on the REE behaviors in porewater of marine sediment^2,21,28^. The records of REE pattern can be used to demonstrate processes of REE cycling in oceans^28^, and the study of REE fractionation and migration between seawater and porewater play a key role for understanding early diagenesis and REE cycling mechanisms^21,28,30–32^.

Porewater which offer a link between bottom water and sediments is a carrier of the chemical components, and act as sensitive indicators of diagenesis^33^. There are few works about porewater profiles for REE due partially to analytical difficulties^30,31,33,34^. Existing REE data of porewater are mainly from shallow sea region^2,31,33,35–37^, and margin and slope facies^2,28^. However, there are still few investigations for REE cycling in deep-sea basin, and in the porewater of pelagic sediment. In addition, deep-sea mud in the Pacific Ocean has been considered as an important potential resource for REE^1^. Researchers of REE-rich sediments mainly focused on enrichment mechanism^38–41^ notwithstanding the fact that investigating REE cycle in deep ocean basin may be also important for uncovering the origin of REE-rich sediments.

In this paper, high-resolution REE profiles in both seawater and porewater are presented for 2 sites in the western Pacific basin. The object is to achieve an improved understanding of (1) features of REE migration and fractionation, and (2) the REE cycling mode in deep-sea area in the western Pacific.

**Figure 1:**
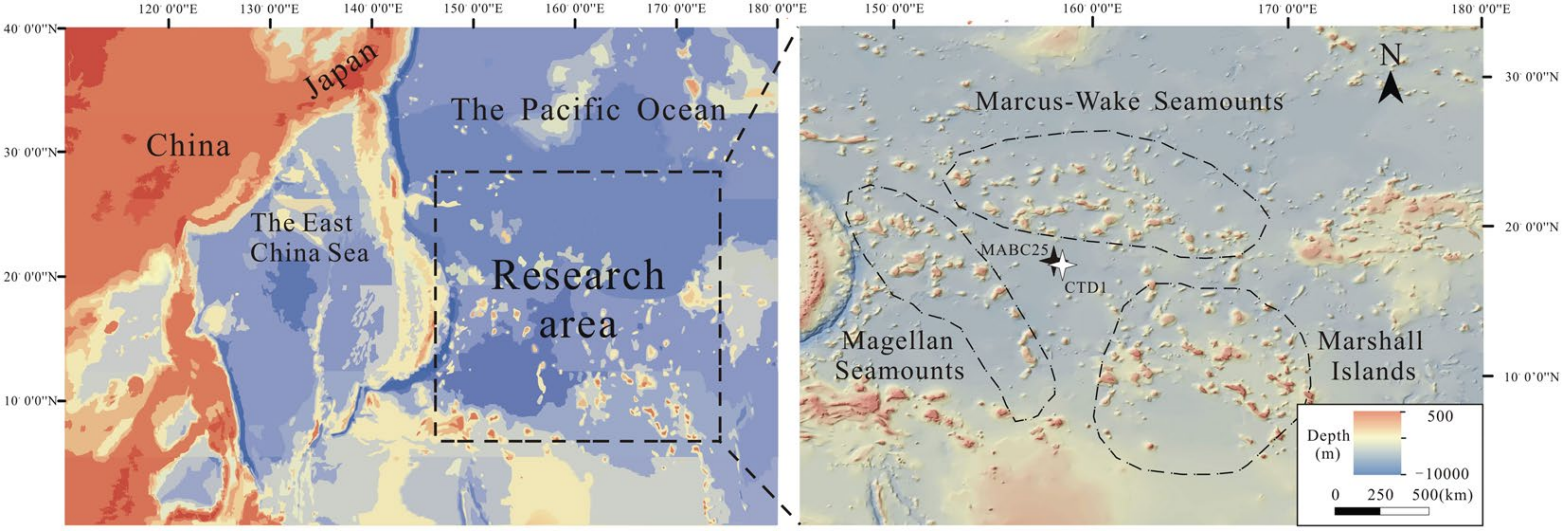
Location of the research area in the western Pacific region.

A comfortable succession of marine sediments was deposited in Pigafetta basin of the western Pacific surrounded by seamounts, with the depth of water ranging from 5000 m to 6500 m (Fig. 1). The seawater oxygen minimum zone (OMZ) ranges from 600 m to 1000 m with oxic bottom water, and the nutrients (SiO_3_^2−^, NO_3_^−^ and PO_4_^3−^) show the low values at surface water followed by a steep rise at OMZ and deep seawater^42^. The Pigafetta basin is typical of pelagic sediment having no abundant riverine input and low values of terrigenous, organic contents and sedimentation rate^43^ compared to the margin and slope sediments^2,28^. These sediments are dominated by zeolitic brown clay in the depth of 0–40 m (equivalent to the Late Cretaceous to the Pliocene)^43^, and show REE enrichment characteristics^1^ with the highest concentration exceeding 6500 ppm near the area^38^. Seawater samples collected at CTD1 site (water depth ~5688 m) and aliquots of 32-cm-thick sediments were collected by box core (MABC25) at water depth ~5322 m in July 2015. The upper part of site (shallow sediments, ~0–8 cm) contains sandy clay with gravel, with high moisture content, low mobility and low viscosity. Whereas the lower part (deep sediments, ~8–32 cm) are dominated by pelagic clay with low moisture content and relatively high viscosity.

## Results and Discussion

### Vertical profiles of REE, Mn, Fe and sulfate in seawater and porewater

**Figure 2:**
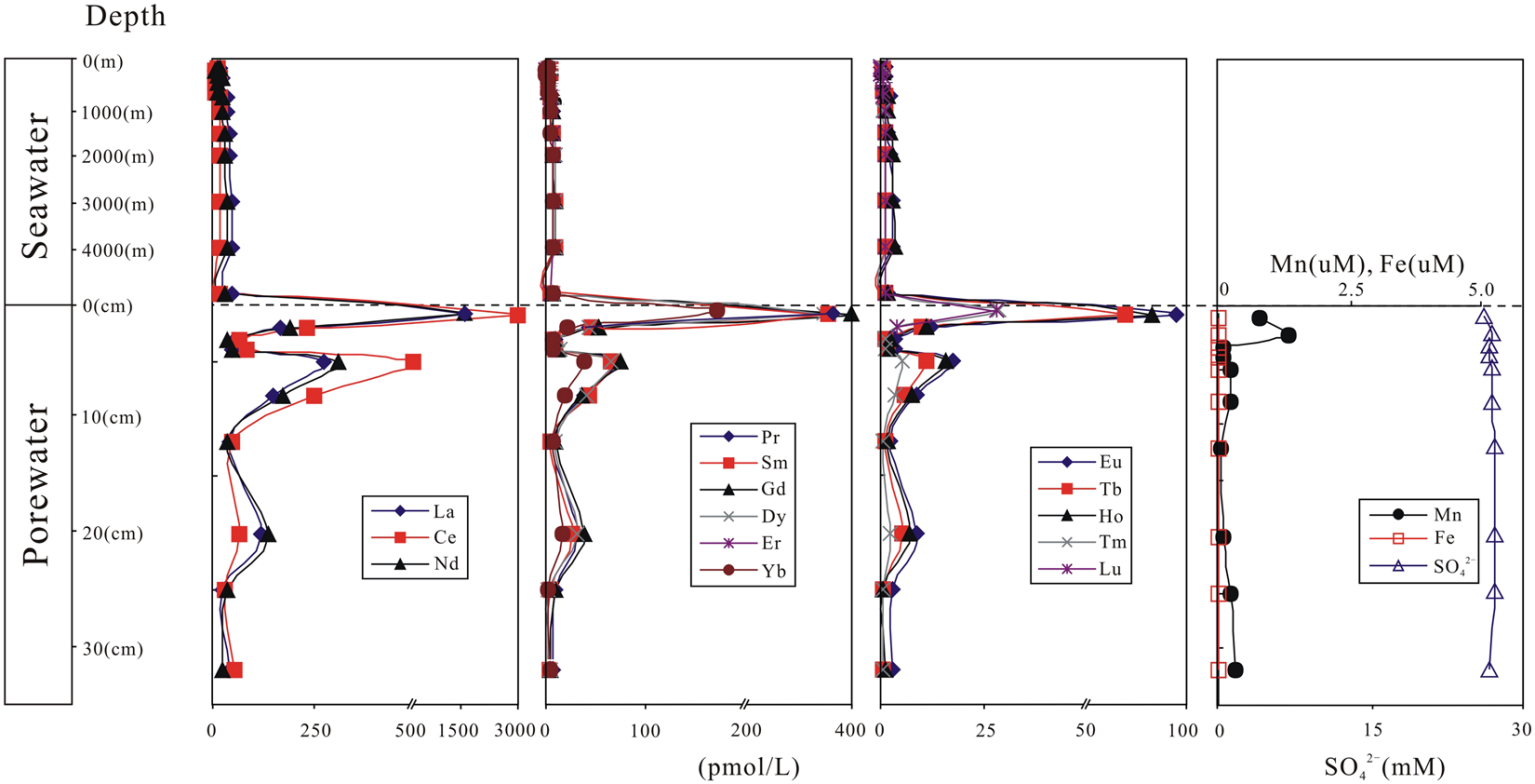
Seawater profile of REE and porewater profile of REE, Mn, Fe and SO_4_^2−^, Western Pacific.

**Figure 3:**
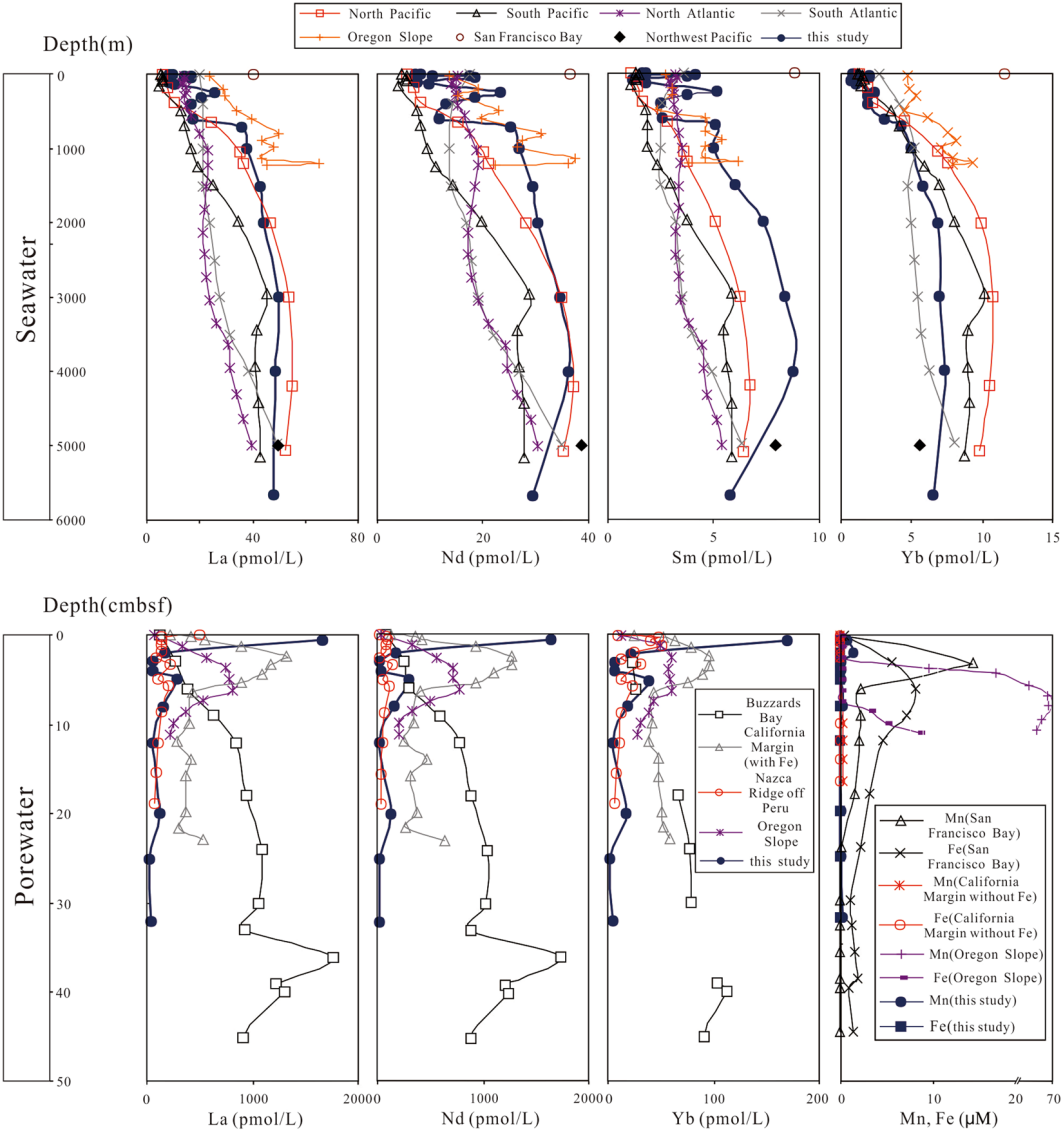
The distributions of REE concentrations from seawater, and variations of REE, Mn and Fe values in porewater. Data source: North Pacific, South Pacific, North Atlantic, South Atlantic, Northwest Pacific, San Francisco Bay from refs.^44–49^ respectively, Oregon Slope and Buzzards Bay from ref.^28^ and ref.^33^ respectively, California Margin and Nazca Ridge off Peru from ref.^2^.

The seawater, porewater and sediment results are presented in the Tables 1, 2 and 3, and Fig. 2. The contents of REE (ΣREE) are low in seawater (31.8–168 pmol/L) and display an increasing trend with depth (Fig. 2), similar to previously published REE data^28,44–49^ (Fig. 3). The increasing trend of REE concentrations in seawater may be caused by many factors. REEs in upper seawater column are removed by adsorption on downward settling particles followed by dissolution of REEs from these particles upon settling into deep seawater^44^. Alternatively, this REEs concentrations gradients may be produced by the REEs-rich upward flux from porewater into bottom water^3^.

**Table 1:** REE geochemical characteristics of seawater in research area, western Pacific.

Sample	Depth	La	Ce	Pr	Nd	Sm	Eu	Gd	Tb	Dy	Ho
	(m)	(pmol/L)									
CTD1-1	3	8.57	6.23	1.67	7.21	1.63	0.39	1.62	0.22	1.60	0.36
CTD1-2	10	9.68	7.85	2.06	7.94	1.81	0.34	1.76	0.28	1.74	0.43
CTD1-3	20	14.0	11.6	3.00	10.5	1.85	0.46	2.41	0.39	2.46	0.51
CTD1-4	30	16.6	10.1	4.15	17.5	4.19	0.99	4.61	0.62	4.03	0.88
CTD1-5	50	16.3	11.6	4.19	18.3	3.81	1.01	4.50	0.70	4.03	0.85
CTD1-6	100	8.34	6.43	1.74	7.32	1.19	0.28	1.53	0.19	1.38	0.31
CTD1-7	125	10.3	6.64	2.32	9.77	1.80	0.48	2.41	0.34	2.22	0.51
CTD1-8	250	25.9	19.3	5.78	23.3	5.20	1.22	5.31	0.84	5.09	1.03
CTD1-9	300	20.7	14.3	4.73	18.2	3.85	1.03	4.34	0.65	3.90	0.84
CTD1-10	400	16.4	8.82	3.17	12.9	2.54	0.65	3.29	0.50	3.23	0.82
CTD1-11	600	17.4	5.69	2.99	11.6	3.06	0.82	3.52	0.73	3.83	1.11
CTD1-12	700	36.1	16.0	8.27	25.0	5.16	1.57	8.50	1.24	7.95	1.68
CTD1-13	1000	38.0	20.8	6.45	26.8	5.05	1.23	6.04	0.90	5.83	1.64
CTD1-14	1500	42.9	16.4	7.51	29.2	6.10	1.71	8.37	1.25	7.80	2.11
CTD1-15	2000	44.2	18.1	8.42	30.3	7.47	1.86	8.10	1.30	8.69	2.02
CTD1-16	3000	50.2	17.3	9.57	34.4	8.49	1.90	9.21	1.26	9.88	2.23
CTD1-17	4000	48.9	18.3	10.0	36.1	8.89	2.04	9.65	1.20	10.4	2.06
CTD1-18	5663	48.1	15.2	7.86	29.3	5.88	1.62	7.03	1.15	7.28	1.88
**Sample**	**Er**	**Tm**	**Yb**	**Lu**	**ΣREE**	**La**_**N**_ **/Yb**_**N**_	**La**_**N**_ **/Sm**_**N**_	**Sm**_**N**_ **/Yb**_**N**_	MREE/MREE*	Ce/Ce*	Eu/Eu*
	**(pmol/L)**										
CTD1-1	1.11	0.12	0.87	0.17	31.8	0.59	0.71	0.83	0.34	0.38	1.12
CTD1-2	1.34	0.17	1.00	0.16	36.5	0.57	0.72	0.80	0.32	0.41	0.88
CTD1-3	1.66	0.18	1.32	0.19	50.5	0.63	1.01	0.62	0.29	0.41	0.99
CTD1-4	2.62	0.34	1.99	0.29	68.9	0.49	0.53	0.93	0.43	0.28	1.04
CTD1-5	2.44	0.31	2.00	0.29	70.4	0.48	0.57	0.84	0.41	0.32	1.11
CTD1-6	1.00	0.12	0.69	0.10	30.6	0.71	0.94	0.76	0.29	0.39	0.95
CTD1-7	1.60	0.24	1.07	0.18	39.9	0.57	0.77	0.74	0.36	0.31	1.05
CTD1-8	3.30	0.45	2.40	0.44	99.5	0.64	0.67	0.96	0.35	0.36	1.08
CTD1-9	2.37	0.31	1.87	0.30	77.4	0.66	0.72	0.91	0.35	0.34	1.15
CTD1-10	2.53	0.35	1.94	0.32	57.5	0.50	0.87	0.58	0.35	0.28	1.02
CTD1-11	3.15	0.65	3.06	0.72	57.7	0.34	0.76	0.44	0.41	0.18	1.15
CTD1-12	4.84	0.70	4.22	0.68	135	0.51	0.94	0.54	0.42	0.21	1.04
CTD1-13	5.11	0.83	4.96	1.01	125	0.45	1.01	0.45	0.30	0.30	1.01
CTD1-14	6.28	0.95	5.80	1.23	138	0.44	0.94	0.47	0.37	0.21	1.07
CTD1-15	6.97	0.99	6.79	1.16	148	0.39	0.79	0.49	0.38	0.22	1.10
CTD1-16	7.12	1.02	6.90	1.19	164	0.43	0.79	0.54	0.39	0.18	0.99
CTD1-17	7.16	1.00	7.00	1.08	168	0.41	0.74	0.56	0.40	0.19	1.01
CTD1-18	5.90	0.93	6.09	1.09	139	0.47	1.10	0.43	0.32	0.18	1.15

**Table 2:** Geochemical characteristics of porewater in MABC25, western Pacific.

Sample	Depth	La	Ce	Pr	Nd	Sm	Eu	Gd	Tb	Dy	Ho	Er	Tm
	(cm)	(pmol/L)											
MABC25-1	0-1	1670	3059	384	1654	362	92.5	395	63.8	351	72.1	203	29.0
MABC25-2	1-2	167	236	43.9	190	46.7	12.3	53.2	9.73	44.5	11.3	26.7	3.86
MABC25-3	2-3	57.0	65.8	8.61	36.8	7.78	3.59	10.4	1.36	12.1	2.44	7.25	0.94
MABC25-4	3-4	49.5	84.7	10.8	46.1	9.28	3.42	11.3	1.64	14.5	2.52	8.01	0.97
MABC25-5	4-5	275	502	70.4	311	65.9	17.7	75.1	11.2	64.7	15.9	42.0	5.53
MABC25-6	5-8	148	253	38.7	174	42.8	8.88	36.6	5.98	40.9	7.82	24.6	3.39
MABC25-7	8-12	41.5	49.1	7.65	33.3	4.69	2.37	9.61	1.12	9.63	1.56	6.84	0.83
MABC25-8	12-20	119	63.5	31.6	139	26.3	8.78	39.1	5.55	32.4	6.97	21.0	2.60
MABC25-9	20-25	22.3	27.8	8.94	34.0	3.76	2.71	9.31	0.75	3.34	0.86	3.40	0.38
MABC25-10	25-32	39.7	55.5	6.27	23.6	3.07	3.21	5.10	0.69	5.55	1.24	4.17	0.74
**Sample**	**Yb**	**Lu**	**ΣREE**	**LaN/YbN**	**LaN/SmN**	**SmN/YbN**	MREE/MREE*	Ce/Ce*	Eu/Eu*	**Mn**	**Fe**	**S04** ^2**−**^	
	**(pmol/L)**									**(umol/L)**		**(mmol/L)**	
MABC25-1	170	27.4	8532	0.58	0.62	0.94	0.28	0.88	1.13	0.77	b.d.^a^	26.06	
MABC25-2	22.4	3.84	871	0.44	0.48	0.92	0.39	0.63	1.13	1.34	b.d.	26.68	
MABC25-3	7.67	1.21	223	0.44	0.98	0.45	0.31	0.67	1.80	0.11	b.d.	26.47	
MABC25-4	7.54	1.45	252	0.39	0.72	0.54	0.31	0.85	1.52	0.077	b.d.	26.50	
MABC25-5	39.4	6.29	1502	0.41	0.56	0.74	0.30	0.83	1.15	0.22	b.d.	26.79	
MABC25-6	19.9	3.08	809	0.44	0.47	0.95	0.33	0.77	1.04	0.25	b.d.	26.85	
MABC25-7	6.30	0.93	175	0.39	1.19	0.33	0.30	0.63	1.47	0.034	b.d.	27.12	
MABC25-8	17.8	2.28	516	0.40	0.61	0.65	0.46	0.24	1.22	0.084	b.d.	27.03	
MABC25-9	2.85	0.27	121	0.46	0.80	0.58	0.32	0.44	1.83	0.22	b.d.	27.04	
MABC25-10	5.51	0.91	155	0.43	1.74	0.25	0.21	0.80	3.53	0.30	b.d.	26.58	

^a^b.d. = below detection.

**Table 3:** REE and trace elements concentration of sediment in MABC25, western Pacific.

Sample	Depth	La	Ce	Pr	Nd	Sm	Eu	Gd	Tb	Dy	Ho
	(cm)	(ppm)									
MABC25-1	0	86.5	112	22.5	101	22.4	5.03	24.1	3.73	21.7	4.82
MABC25-2	8	102	120	25.6	115	27.2	5.83	28.8	4.61	27.6	6.42
MABC25-3	15	95.4	108	24.8	112	24.8	5.40	26.7	4.25	25.3	5.90
MABC25-4	20	112	124	27.6	123	28.6	6.01	29.9	4.86	29.2	6.86
MABC25-5	32	103	113	26.4	119	26.9	6.15	28.8	4.62	28.4	6.50
**Sample**	**Er**	**Tm**	**Yb**	**Lu**	**ΣREE**	**U**	**Ba**	MREE/MREE*	**Mn**	**Al**	**Mn/Al**
	**(ppm)**								**(%)**		
MABC25-1	12.8	1.86	11.6	1.73	432	2.35	863	0.35	0.60	8.23	0.072
MABC25-2	16.7	2.38	15.1	2.28	500	2.36	867	0.38	0.55	8.93	0.062
MABC25-3	15.3	2.17	13.8	2.06	466	2.31	583	0.37	0.75	8.42	0.089
MABC25-4	18.1	2.56	16.0	2.41	531	2.40	428	0.37	0.85	8.57	0.099
MABC25-5	16.8	2.31	14.8	2.18	498	2.61	555	0.38	0.79	8.56	0.092

For comparison, La, Nd, Sm and Yb seawater column profiles from previously researches are shown in Fig. 3. As can be seen, the content variations revealed in our results are comparable to the North Pacific, particularly for La and Nd. In the upper seawater, nutrients are assimilated by phytoplankton, which is remineralized and released REE back into solution, resulting in low concentrations at the surface water but a rapidly increase in the oxygen minimum zone (OMZ)^50^. The OMZ (~600–1000 m) in study area shows significant enrichment of nutrients (nitrate, phosphate and silicate)^42^ and REEs (Fig. 3), and thus suggests that REEs are influenced by biogeochemical processes in shallow water. However, advective transport and water mass mixing may be the dominant process controlling REEs in the intermediate and deep ocean^47^. It was established that REEs concentrations increase in deep water from North Atlantic to North Pacific^45^ (Fig. 3). Data in this study show relatively high values of Sm and low concentrations of Yb in deep water compared to North Pacific (Fig. 3), and this observation might probably have resulted from different water mass. The most obvious influence on REE in the western Pacific is the northward spreading Antarctic bottom water (AABW)^44^. Kawabe *et al*.^51^ inferred that these water mass is mixed with North Pacific Deep Water (NPDW) in study area. Therefore, the striking similarity in REE concentrations between deep water in Northwest Pacific nearby research area and the deep-water samples of CTD1 are observed (Fig. 3).

REE concentrations in porewater are relatively high, with the peak value at sediment-water interface (SWI). Total concentrations of REE range from 120 pmol/L to 8531 pmol/L with relatively high abundances in upper part of the core (Fig. 2).

REE contents (except the top sample) show relatively low values similar to Nazca Ridge off Peru (Fig. 3) which exhibits no Fe increase in porewater^2^. It was suggested that high concentrations of REEs in porewater are derived from reduction of oxides in reducing condition^2,28,33^, which is supported by the strong increase of dissolved Mn and Fe at the upper part of sediments at each site (Fig. 3). However, there is extremely low Fe concentrations (below detection limit) and weak enrichment of Mn in this study coinciding with California Margin site (without Fe present) (Fig. 3), which indicates that the studied interval of the MABC25 profile is oxic or suboxic. This interpretation is also supported by the sulfate concentrations that are close to typical seawater value of ~28 mmol/L^52^ for all porewater samples (Fig. 2) and no obvious excursion in Ba for sediment samples (Table 3). Mn/Al ratios in bulk sediment are used as a sensitive indicator of suboxic condition, due to manganese is soluble under suboxic condition in porewater and transport upward until oxic zone^53^. Consequently, sediments under suboxic condition exhibit low Mn/Al value of ~0.01^52^. The Mn/Al ratios in MABC25 sediments range from 0.062 to 0.099, thus showing much higher values than the value of ~0.01 which is suggestive of oxic sedimentation throughout. This interpretation is also evidenced by extremely low U concentrations of bulk rock (2.31~2.61 ppm)^52^. The dissolved Mn of porewater values correspond to results of ocean drilling program which show seawater-like value 22 m below seafloor (mbsf) and peak value (~86 μmol/L) at 232 mbsf in research area^43^.

There are obvious REE enrichment at SWI (discussed below) and weak enrichment of dissolved Mn at a depth of ~0–2 cm below the seafloor (cmbsf). The characteristics of dissolved Mn may be in contradiction to oxic condition in porewater. Reconciling this conflict, Kim *et al*.^54^ and Machida *et al*.^55^ observed that the geochemistry of ferromanganese nodule on deep-sea sediment is similar to that of the adjacent Fe-Mn crust on the seamounts nearby the research area, thus indicating a high flux of detrital components of Fe-Mn crust from seamounts to the study site. The increase of dissolved Mn at top sediment is probably caused by interference of partial dissolution of these detrital materials. The detrital grains, remarkable peak of dissolved Al concentration, which mainly comes from detrital components and positive correlations between dissolved Al and trace elements, as observed in upmost sediments of MABC25 site^42^, supporting this interpretation.

### Comparison between seawater and porewater REE distribution patterns

**Figure 4:**
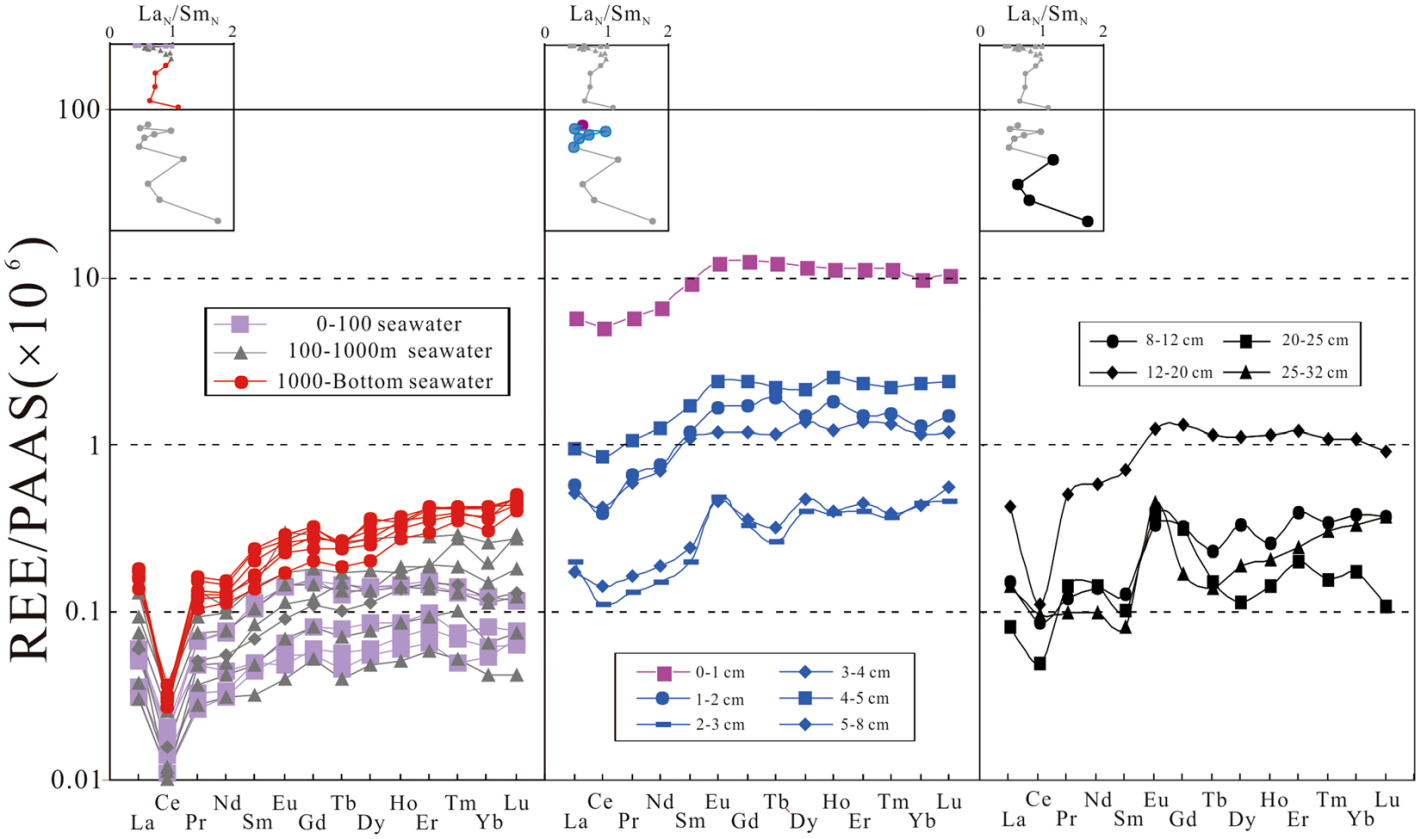
PAAS shale-normalized REE distribution spectra of seawater and porewater.

The PAAS-normalized patterns of seawater samples display the typical characteristics of seawater^3^, with a progressive enrichment toward heavy REE, a depletion of Ce and a slight enrichment of Gd (Fig. 4). Specifically, the intermediate and deep seawater column (~1000–5663 m) exhibits a stronger enrichment of HREE, and a stronger depletion of Ce (with an average Ce/Ce* value of 0.19) than those of the upper seawater column (~0–1000 m) (Fig. 4).

The “MREE bulge” pattern was observed in porewater with anoxic condition^2^. Porewater REE patterns in this study, however, show a slight decline to left and negative Ce anomalies (Fig. 4), also reflecting oxic condition in the sedimentary profile of the western Pacific, which is in consistent with the central Pacific^56^ and the eastern Pacific area^57^. The REE patterns of porewater can be divided into two patterns. Porewater REE patterns of the upper part of core (shallow porewater, ~0–8 cm) show a flatter pattern with weak negative Ce anomalies and the enrichments of LREE and MREE relative to bottom seawater. The lower part of the porewater (~8–32 cm), however, shows stronger positive Eu anomalies and negative Ce anomalies (Fig. 4).

### Migration and fractionation of REE

**Figure 5:**
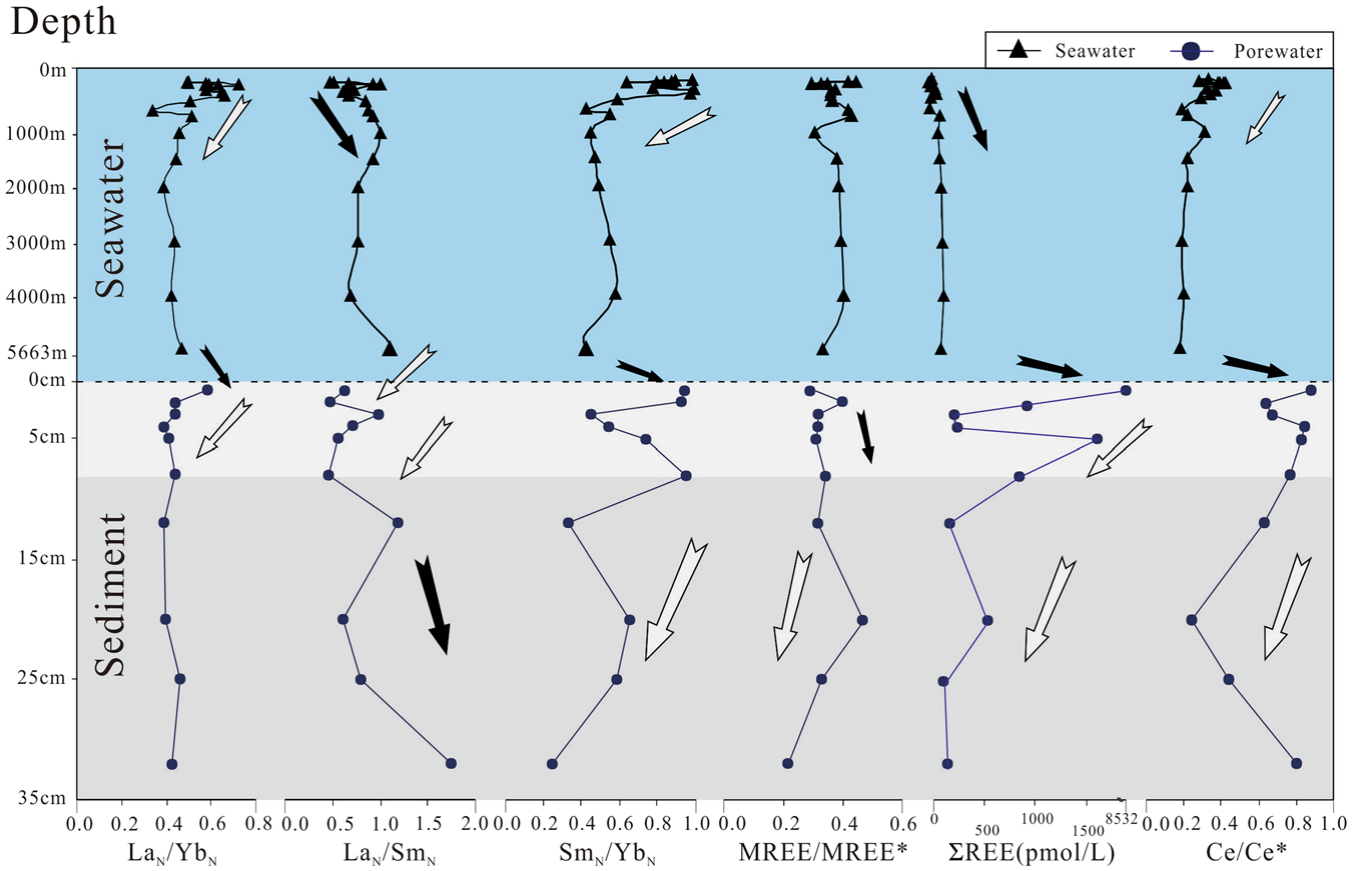
Variation characteristics of REE in seawater and porewater.

The ratios between LREE (La-Nd), MREE (Sm-Dy) and HREE (Ho-Lu) are expressed as La_N_/Yb_N_, La_N_/Sm_N_ and Sm_N_/Yb_N_ respectively. REE contents in the surface seawater is low and La_N_/Yb_N_, La_N_/Sm_N_ and Sm_N_/Yb_N_ values are 0.59, 0.71 and 0.83 respectively (Fig. 5), indicating a relative HREE enrichment. The surface seawater also shows a flat REE pattern and a relatively weak Ce depletion (Ce/Ce* = 0.38). When the main contribution of REE is river or eolian input, the PAAS-normalised REE would exhibit a shale-like flat pattern with La_N_ ≈ Ce_N_ ≈ Pr_N_ ≈ Lu_N_^10,31,58,59^. Organic matter, Fe and Mn particle scavenged abundant REEs from seawater with adsorption capacity of LREE > MREE > HREE^2^. Therefore, surface seawater usually shows a HREE enriched pattern and a relatively flat distribution in comparison with deep seawater^2^.

The values of La_N_/Yb_N_ and Sm_N_/Yb_N_ show obvious decline and La_N_/Sm_N_ ratios rise at depth of OMZ^42^ (~600–1000 m), followed by nearly constant values of these ratios in deep water (~1000–5663 m), reflecting significant enrichment of HREE and depletion of LREE and MREE at intermediate and deep seawater (Fig. 5). These results are consistent with data from the northern Atlantic^3^, the South Atlantic^47^, the South Pacific^45^ and the North Pacific^44^. The variation of REE fractionation may be produced by adsorption and the strength of complexation. Deep seawater displayed a significantly HREE-enriched pattern due to preferential scavenging of LREE and MREE by particles, and stronger compelxation of HREE in seawater^23,60^. The deep-water samples which are influenced by biochemical processes and water mass mixing^47^ show constant REE contents and distribution patterns (Figs 4 and 5), indicating a stable state. The upward porewater flux can lead to REE enrichment in deep water^3^. The ΣREE of bottom seawater sample, however, exhibit low value relative to surface porewater and even lower than the seawater sample at depth of ~4000 m, indicating that the diffusion of flux from sediments is low. This result is consistent with the suggestion of Piepgras and Jacobsen^44^. They also observed no significant upward REEs flux from the oxic sediments. Cerium (Ce) exists as trivalent or tetravalent forms depending on the redox conditions^2,13,15,61,62^. Ce/Ce* values decrease with water depth, suggesting that surface seawater is influenced by REEs sources with LREE enrichment and higher Ce/Ce* values. Deep seawater, however, may be influenced by oxic AABW^44^, with low Ce/Ce* values due to Ce scavenging by non-dissolve oxide.

Porewater at SWI (~0–1 cm depth of sediment) shows a significant REE enrichment, with concentrations almost hundred times higher than surface seawater as a result of particles degradation during early diagenesis^2,28,33^. There are abundant particles in the surface water but few particles in deep seawater in research area (unpublished data). Although, Müller *et al*.^57^ inferred that only 1% particles could reach seafloor, these should not be ignored in REE cycling. It is believed that the particles are dominantly in particulate coatings composed of Mn- or Fe-Mn oxides and organic matter, and only Fe-oxides and organic matter coatings are the main carriers of REEs^2^. The dissolved Fe is not present in MABC25 (Fig. 2), thus the REEs may be released by organic matter. Most of organic matter would be expected to degrade within the upper ~1–2 cm^63^, particularly in oxic sediments which shows a markedly faster degradation rate of organic matter relative to anoxic sediments^2^. This interpretation is also evidenced by the low values of total organic carbon (~0.2–0.4 wt%) of shallow sediments in North Pacific^64^ and the extremely low value (0.03 wt%) of sediments in research area^43^. It was established that the organic matter in surface sediments shows non-negligible REE concentrations with an average value of ~39 ppm, and displays a slight decline to right pattern with enrichment of LREE and MREE and no or slight positive Ce anomalies^65^. These results are in agreement with substantial LREE, MREE and Ce scavenging by organic matter in the upper water column^21^. The organic matter mainly originated from plankton in euphotic zone^63^, but εNd values suggesting that there is a detrital provenance in Northwest Pacific^65^. The ΣREE of porewater at SWI show much higher concentrations compared to the published data from oxic surface sediments (Fig. 3), indicating another source of REEs in surface porewater besides organic matter. It is possible that detrital components may play a key role for REEs enrichment. Freslon *et al*.^65^ infer that partial dissolution of easily dissolvable volcanogenic particles could occur in Northwest Pacific. Lots of seamounts around the MABC25 (Fig. 1) and abundant enrichment of dissolved Al at SWI^42^, indicating that dissolution of detrital components is also a reason for the significant increment in ΣREE in surface porewater. The La_N_/Yb_N_ and Sm_N_/Yb_N_ values increase and La_N_/Sm_N_ (Fig. 5) obviously decrease, representing LREE and MREE enrichments. These characteristics are similar to organic matter^65^ and detrital components^2^, indicating that the LREE and MREE carried by organic matter and detrial components have been released into porewater during burial in the sediment surface. The increasing of Ce/Ce* values may also be generated by these processes (Fig. 5).

The shallow porewater (~1–8 cm) show a rapid decline of ΣREE (i.e. mean value of ~731 pmol/L) and the decrease of La_N_/Yb_N_ values (Fig. 5), which may reflect the decrease of REE release from organic matter and detrial components, or the increase of REE removal. The slightly increased MREE/MREE* values may reflect lower activity and thus gradually released of MREE from particles. Similarly, the decreased La_N_/Sm_N_ ratios and increased Sm_N_/Yb_N_ ratios at depths of >3 cm show MREE enrichment of porewater.

REEs of porewater in deeper sediments (~8–32 cm) show very low contents, i.e., ΣREE less than bottom seawater. The decreasing ΣREE values may be influenced by early diagenetic reaction^33^. The REEs may be removed by precipitation of REE-rich mineral or the absorption by sediments^21^. The steep drop of MREE/MREE* and Sm_N_/Yb_N_, and obvious increase of La_N_/Sm_N_ values indicate a severe removal of MREE (Fig. 5). It is possible due to the exhaust of particles and absorption of sediments. The uptake of MREE is further evidenced by increase of MREE/MREE* in sediment samples with depth from 0.35 to 0.38 (Table 3). MREE may be influenced by surface charges of Fe- and Mn- oxyhydroxides. Specifically, Mn- oxyhydroxides tend to display negative charges that attract LREE, but Fe- oxyhydroxides develop positive charges that attract HREE^66^, leading to a preferential scavenging of MREE in porewater by Fe-Mn particles. Alternatively, recent works observed that REEs exhibited a strongly positive correlation with phosphorus^38–41,67,68^, and phosphate minerals in sediments could also uptake abundant MREE^69–71^, resulting in depletion of MREE in porewater. The sequestering of REE has been observed by investigating the partitioning of REE between different reservoirs in research area^41^. Wang *et al*.^41^ calculated that the apatite occupies above 70% of the total REE budget and Fe-Mn micro-nodules and zeolite only occupy 2% and 3% respectively. Thus, REE mainly uptake by apatite in sediments. Ce/Ce* values exhibited gradual decreasing trend in the interval (Fig. 5) with oxic condition. The most reasonable explanation is the preferentially removal of Ce in deeper porewater^33^.

As an element with multiple valences, europium (Eu) exist as trivalent in seawater and would produce positive Eu anomalies under hydrothermal input or strongly reducing conditions^61^. There are no obvious hydrothermal activity and reducing conditions in research site. Thus, the positive Eu anomalies in porewater may result from abundantly uptake of other MREEs by sediments, or interference of Ba related to ICP-MS analytical techniques^72^. However, the seawater-like concentration of sulfate in porewater and low values of Ba in sediments point to the fact that there are low dissolved Ba concentrations in porewater. The low values of Ba interference may reflect the original signature of Eu^62^.

From the discussions above, we can conclude that: (1) surface seawater column displays a relatively flat REE distributions pattern due to influence of the input flux carried by wind or ocean current, (2) deep seawater shows HREE enrichment, and LREE and MREE depletion because of biogeochemical processes and water mass mixing (3) porewater at SWI exhibits REE enrichment (especially LREE and MREE) resulting from degradation of organic matter and dissolution of detrital components, (4) particles begin to release MREE in shallow sediments due to a slow regeneration rate, and (5) porewater in deeper sediments presents a steep fall of REE concentrations and MREE depletion as a result of absorption of sediments.

### Deep-sea REE cycling and their indicative significance

Historically, riverine inputs were considered as the primary source of REEs to seawater^18–20^ and could be transported to marine via particles, solution and colloids with a total concentration of 39 × 10^6^ mol/y^73^. Similarly, the flux of REEs from eolian or atmosphere input also adds abundant REEs into ocean. Hydrothermal with low REE contents are poorly quantified^21^ but likely play a limited role in REE cycle. Recent works found that groundwater could discharge lots of REEs to seawater^29^, and porewater is also thought to be a significant source of REEs to seawater^28,31–33^. Chen *et al*.^21^ predicted that coastal and shelf sediments would diffuse REEs to seawater 10 times higher than river inputs. Deep sea area (i.e. continental margin and slope) also show the same characteristics with Nd from porewater to seawater up to ~110 × 10^6^ mol/y^28^.

**Figure 6:**
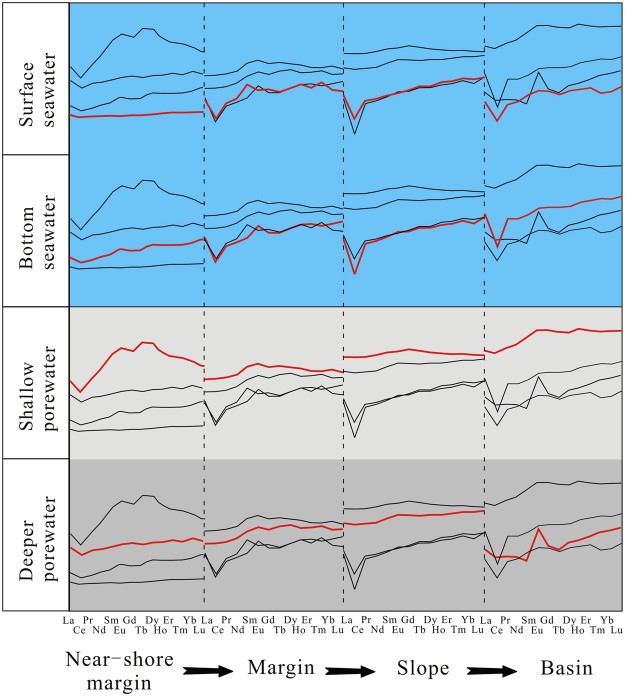
Seawater and porewater PAAS normalized REE patterns, Pacific. Each plot shows all the data for that site in black, and the relevant layer data is shown in red. Data sources: near-shore margin from ref.^2^, margin and slope from ref.^28^, and basin (this study).

There are HREE depleted pattern in shallow porewater, and MREE and HREE enriched pattern (similar to the pattern of deep seawater) in deeper porewater (Fig. 6) at near-shore margin site of the Pacific^2^. This observation is not unique^28^. Abbott *et al*.^28^ observed the same porewater REE patterns in the sediments of margin and slope sites (Fig. 6) and interpreted that HREE depletion patterns for shallow porewater may have a primary diagenetic source. Moreover, the HREE enrichment patterns for deep porewater which displayed a similar REE pattern with bottom seawater, suggesting that porewater in shore margin or slope sediments are the main REE source to seawater^28^. However, our data shows difference REE distribution patterns between bottom seawater and porewater at shallow or deep sediments in the western Pacific basin (Figs 4 and 6). The REE concentration of the surface porewater are much higher than that of the bottom ocean water, suggesting a REE diffusion flux from sediments to seawater. However, their REE patterns are somewhat different, which may be generated by relatively high mixing rate of deep seawater with mean speed values of ~2.8 cm/s detected by current meter in a year (unpublished data) and/or the limited REEs flux from sediments to seawater. This is also corroborated by the obviously different La_N_/Yb_N_, La_N_/Sm_N_ and Sm_N_/Yb_N_ values between bottom water and porewater (Fig. 5). This feature is apparently different with the shore margin or slope model (Fig. 6).

Possible reasons for REE enrichment of deep water sediments in study area are different characteristics of sediments, sedimentation rates and redox condition compared with shallow water regions. The shallow sediments (~0–8 cm) dominated by sandy clay with high moisture content and permeability, and the deeper sediments (~8–32 cm) contains brown pelagic clay with low moisture content and permeability. The sandy clay in shallow sediments mainly influenced by detrital components from seamounts and organic matter input show substantial release of REEs from sediment into porewater and the high permeability contribute to REEs migrate to deep sediments. The deeper pelagic clays characterized by low permeability and superior adsorption abilities for REEs^74^, largely absorbed REEs from shallow porewater. Meanwhile, the low sedimentation rate is favorable for REE enrichment because the ΣREE of sediments is mainly controlled by REE scavenging processes from seawater to sediments^75^. The age of sediments should be no older than Cenozoic, and the sedimentation rate is lower than 0.5 m/m.y. in pelagic clays in research area^43^. The extremely low sedimentation rate results in the great the uptake of REEs during sedimentation. In addition, redox condition also plays a key role for REE enrichment^67^. The shore margin or slope sediments generally display a relative narrow oxic condition, e.g., only 1mm-thick of sediment in Buzzard bay^30^. Under anoxic condition, there is re-mineralization of oxides in sediments and discharge of abundant REEs^2^. Thus, REEs flux from sediments to seawater primarily comes from the re-mineralization flux^21^. In this study, oxic condition are extensive in porewater (discussed above), which indicated low chances of re-mineralization of oxides. Meanwhile, there are likely extensive re-mineralization of organic matter and dissolution of detrital components. These REEs could be absorbed by sediments which exhibit excellent absorbency.

**Figure 7:**
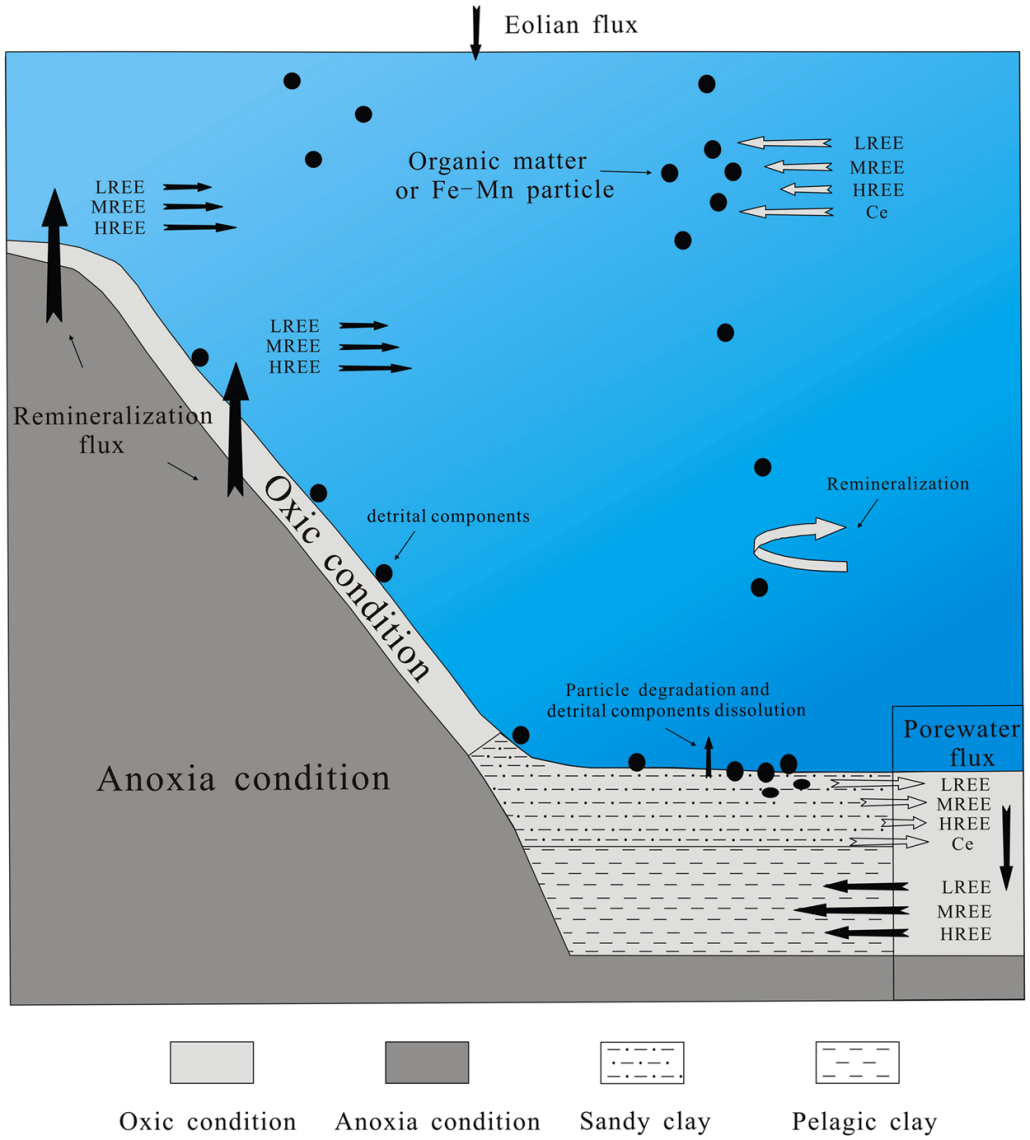
REE cycle model in western Pacific basin.

Arsouze *et al*.^76^ presented Nd oceanic model. This study continues the modeling work and adds the porewater characteristics to establish a possible REE migration and cycle model in the western Pacific basin, which is further compared with REE characteristics of shore margin and slope area (Fig. 7). Two significant REE sources have been proposed (river source is indistinctive in research area), in which eolian sources (LREE ≈ MREE ≈ HREE) input REEs into surface seawater, and re-mineralization porewater flux from shore margin or slope sediments input abundant REEs into bottom seawater (Fig. 7). The particles scavenge REEs from upper water column followed by part re-mineralization in deep seawater. With the settlement of these particles on the seafloor, abundant REEs are released (LREE > MREE > HREE) to porewater at SWI (Fig. 7). The obvious enrichment of REEs in porewater of surface sediments likely leads to part of REEs diffusion to bottom water. However, it may be also reasonable to suppose that abundant REEs are absorbed by the deeper sediments. Preferential uptake of MREE is observed in sediments probably because of existence of apatite. Finally, REEs from upper seawater column are enriched in sediment through a series of migration and fractionation process (Fig. 7).

This model can also account for REE enrichment mechanism. Under anoxia condition, oxides which show enrichment of REEs tend to be regenerated and thus release abundant REEs into seawater. However, sediments with extensively oxic condition in the study area offer a favorable site for oxides preservation. Also, strong adsorption capacity and low sedimentation rate play a key role for REE enrichment. It is worth to remark that pelagic clay in basin displays beneficial preservation condition, low sedimentation rate and excellent absorbency. Thus, we infer that this REE cycle model in deep sea basin is the main reason for widespread enrichment of REEs in sediments of the western Pacific Ocean.

## Conclusions

Surface seawater displays relatively flat patterns due to the eolian sources. The intermediate and deep seawater column showed HREE enrichment patterns because of particles (i.e. Fe-Mn or organic matters) scavenging (LREE > MREE > HREE) and strong carbonate complexation of HREE. The deep water is also influenced by the water mass mixing. The generation of REE-rich particles on the seafloor release abundant REEs (LREE > MREE > HREE) to porewater at the SWI, leading to strong REE enrichment at uppermost porewater with a flat REE pattern. Decomposition present in decreasing trend with depth and part of REEs likely diffuse to bottom ocean water. However, we infer that lots of REEs are absorbed by the deeper sediments. The REE enrichment in basin may be generated by strong adsorption capacities, low sedimentation rate and widespread oxic conditions. We conclude that this mechanism may provide a valuable insight into the REE cycling in the deep sea.

## Methods

### Sampling

The seawater and sediment samples were collected in the ship. All bottles, samplers, tubing and filters were cleaned by acid and ultrapure water before use. Seawater was collected by Sea-Bird Electronics 917 Plus CTD (Conductance, Temperature and Depth) device. Niskin sampling bottles were deployed on an epoxy-coated CTD rosette attached to a non-metallic line in order to minimize chemical contamination, and were programmed to trip at determined depths (bottom water 25 m above seafloor). About 1 L seawater per sample was filtered (0.4 μm) and transferred into polyethylene bottle immediately after sampling. These samples were acidified to pH of 2 with high purity HCl.

Sediment samples were obtained using box core during the Haiyang-6 Cruise by the Guangzhou Marine Geological Survey (GMGS). Within two hours after sediment collection, high-resolution porewater samples were extracted from sediments via Rhizon CSS (Rhizosphere Research Products, Netherlands) moisture sampler which was composed of core solution sampler and syringe. This sampler made of a hydrophilic porous polymer tube, with pore diameter of 0.1 μm, extended with a polyvinyl chloride tube. The outer diameter of a Rhizon is 2.4 mm, and the filter section has a length of 5 cm. Rhizons can minimize sampling artifacts and obtain good results of pH, alkalinity, sulfate, and various metal ions in porewater^77^. Immediately following sampling, the porewater samples were acidified to pH 2 with ultra pure HCl and subsequently transferred into the polyethylene tubes. The seawater, porewater and sediment samples were stored in seal and cool condition (4 °C).

### Analytical methods

Dissolved REE concentrations were measured following the main procedures described in Akagi *et al*.^78^ and Freslon *et al*.^79^. To increase the accuracy of these measurements, a condensation procedure via the co-precipitation was applied to the seawater and porewater samples. Briefly, 25 ml of sample was mixed in a pre-cleaned Teflon centrifuge tube with 0.5 ml of 20 mg/L Ga standard solution. The mixture was shaken and then allowed to equilibrate for 15 min. The solution was increased pH to ~9.0 using 2 M NaOH, which led to co-precipitation of magnesium hydroxides with gallium. The sample was centrifugated for 5 min at 3000 rpm and the supernatant discarded. Following the process of centrifugation was conducted three times, the residue of precipitate (the pre-concentration factor of up to ~200)^78^ was washed by ultrapure water and then redissolved in 4 ml 5% ultrapure diluted HNO_3_ in cleaned Teflon centrifuge tube. Rare earth elements were measured via ICAP-Q Inductively Coupled Plasma Mass Spectrometry (ICP-MS) (Thermo Fisher Scientific, MA, USA) at Third Institute of Oceanography, State Oceanic Administration, China.

For the procedural blanks, the reagent blanks caused by the whole procedure were determined by carrying out the same pre-concentration procedure without use of a seawater or porewater sample. The blanks varied between 0.041 (for Tm) and 3.60 (for La) pmol/L and the procedural detection limit (three times the standard deviation of 10 blank measurements) range from 0.012 (for Tm) to 0.98 (for La) pmol/L (Table 4). Due to no certified reference materials (CRM) for porewater available, seawater CRM (NASS-6) were used to assess the accuracy (ten repeated analyses). The analytical results for every REE agreed with literature values^80^ (Table 4). The external accuracy was assessed via repeat analyses of a porewater sample. The relative standard deviation (RSD) for every REE in samples was ≤10% (seven repeated analyses MABC25-5) (Table 4). The recovery from the column was evaluated using 100 ng/L REE multi-element standard solutions and the sample mean recoveries range from 93% to 102%.

**Table 4:** Blank, limit of detection and results for NASS-6 and MABC25-5 (values in pmol/L), NASS-6 data from ref.^80^.

REE	Blanks	Detection limit	NASS-6			MABC25-5	
			Measured	RSD %	Reference	Measured	RSD %
La	3.60	0.98	83.9	3.5	91.4	275	4.0
Ce	2.86	0.68	45.5	5.5	44.3	502	6.4
Pr	0.71	0.17	11.1	8.9	14.2	70.4	6.0
Nd	2.78	0.18	48.1	9.3	41.6	311	1.2
Sm	0.67	0.18	6.38	5.3	5.39	65.9	5.9
Eu	0.13	0.063	1.97	9.3	1.58	17.7	9.1
Gd	0.51	0.11	6.17	8.7	6.36	75.1	9.9
Tb	0.075	0.031	1.57	4.7	1.26	11.2	9.1
Dy	0.56	0.15	10.5	8.6	9.85	64.7	5.8
Ho	0.12	0.030	2.30	6.3	1.88	15.9	6.8
Er	0.36	0.12	9.38	7.2	10.2	42.0	5.7
Tm	0.041	0.012	1.24	7.0	1.42	5.53	10
Yb	0.29	0.061	7.80	9.5	7.51	39.4	7.7
Lu	0.046	0.015	1.31	7.3	1.49	6.29	5.3

The concentrations of SO_4_^2−^ were measured by using a 790 Personal IC (Metrohm, Herisau, Switzerland) ion chromatograph equipped with a conductivity detector and an anion exchange column (Metrosep A Supp 4). Sulfate concentrations were determined on 500 folds diluted with ultrapure water. A bicarbonate solution (i.e. 1.8 mmol/L Na_2_CO_3_ + 1.7 mmol/L NaHCO_3_) was used as the eluent and the analytical precision estimated to be <1%. Dissolved Fe and Mn of porewater were determined on 50 folds diluted with ultrapure water followed by the method of Hu *et al*.^81^ and analyzed using the ELEMENT XR High Resolution ICP-MS (Thermo Fisher Scientific, MA, USA). The analytical reproducibility and precision was better than 10%. The SO_4_^2−^, Fe and Mn of porewater were analyzed in the Center for Marine Geochemistry Research at the Nanjing University.

Sediment samples were dried, crushed and pulverized (200 mesh). Major element, trace element and REE analyses of sediment followed the method of Chen *et al*.^82^. Mn and Al were analyzed using AXIOSX X-ray fluorescence spectrometry (PANalytical B. V., Almelo, The Netherlands) at the Key Laboratory of Marine Mineral Resources, Ministry of Land and Resources, China. Overall analytical precision and accuracy were better than 3%. For the REE and trace element analyses, the samples were dissolved using an HCl, HF, and HClO_4_ acid mix, and rhodium was added as an internal standard for concentrations calculation. The REE and U were measured using a  X2 ICP-MS (Thermo Fisher Scientific, MA, USA), and Ba was determined by using Optima 4300DV, Inductively Coupled Plasma Optical Emission Spectrometer (PerkinElmer, MA, USA) at the Key Laboratory of Marine Mineral Resources, Ministry of Land and Resources, China. Analytical precision for elemental concentrations was generally better than 5%.

REE concentrations were normalized to PAAS^83^. Ce/Ce* = Ce_N_/(1/2La_N_ + 1/2Pr_N_), Eu/Eu* = Eu_N_/(1/2Sm_N_ + 1/2Gd_N_) were calculated^50^ (Bau and Dulski, 1996) and MREE/MREE* = 2*average (MREE)/average (LREE) + average (HREE) was calculated according to Chen *et al*.^21^.

### Data Availability

The datasets analysed during the current study are available from the corresponding author upon reasonable request.
